# Supplementary material for: Genomic and Chemical Profiling of B9, a Unique Penicillium Fungus Derived from Sponge
Source: J Fungi (Basel). 2022 Jun 29;8(7):686. doi: 10.3390/jof8070686 (PMC9319512; doi:10.3390/jof8070686)
Supplement: Supplementary file 1 [file jof-08-00686-s001.zip › jof-1785557-supplementary materials.pdf]

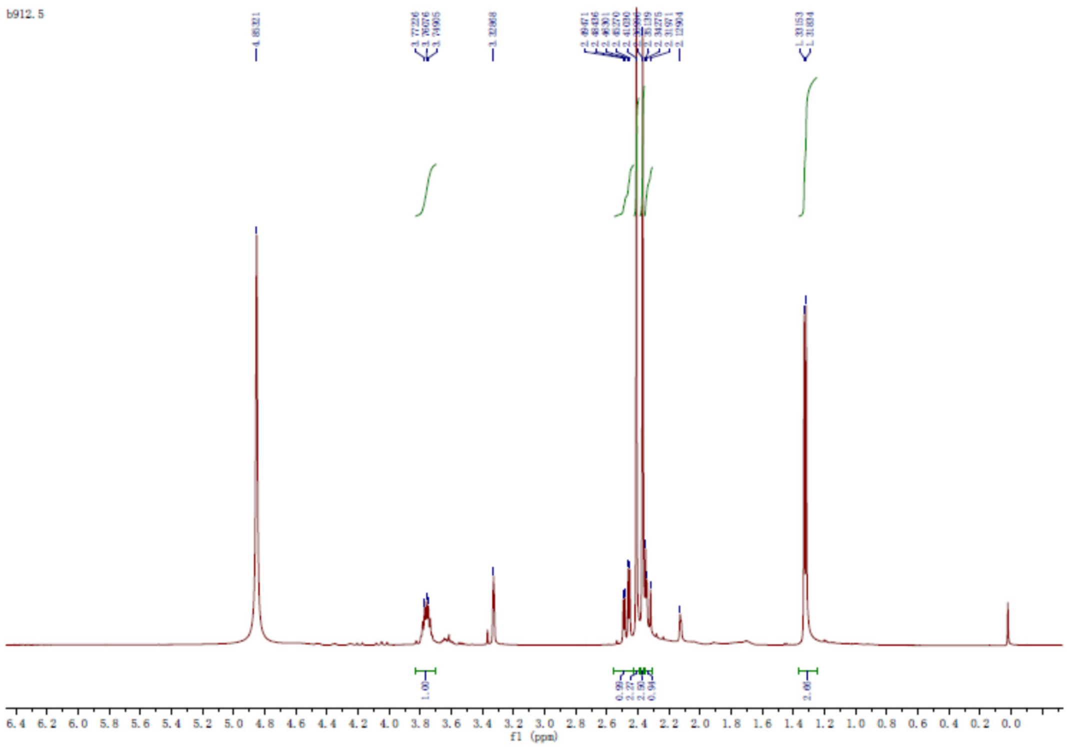

Figure S1. ^{1}H NMR spectrum of compound 2（500 MHz, CD_3OD）

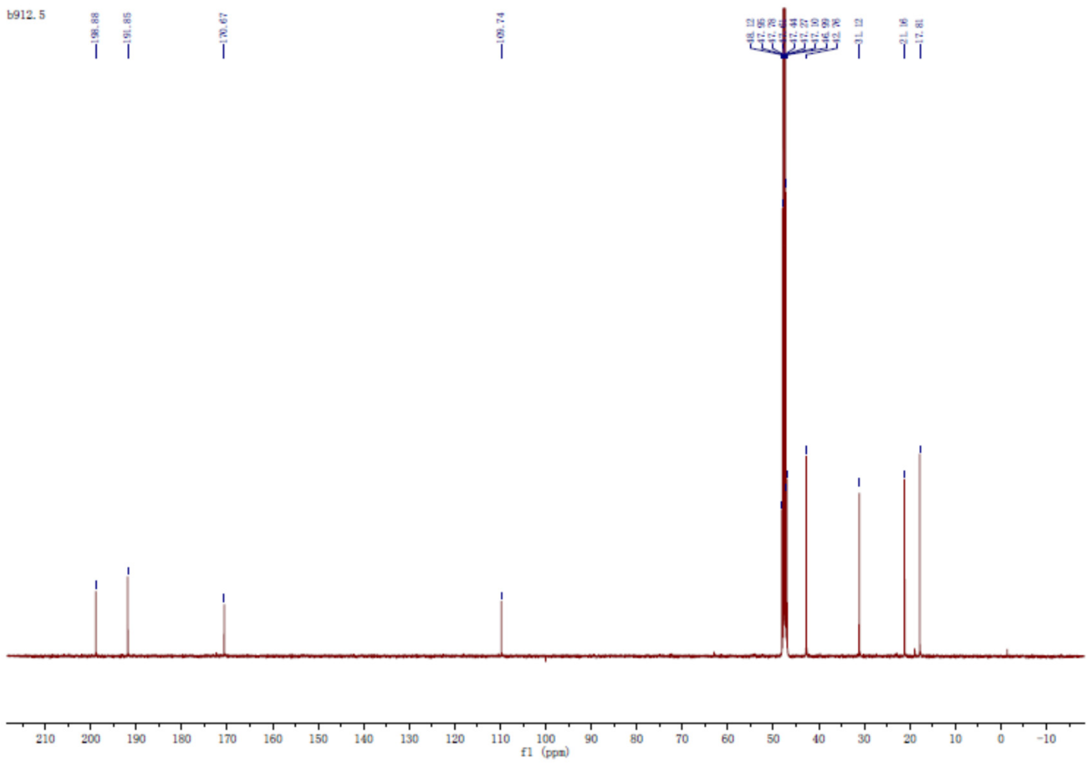

Figure S2. ^{13}C NMR spectrum of compound 2（125 MHz, CD_3OD）

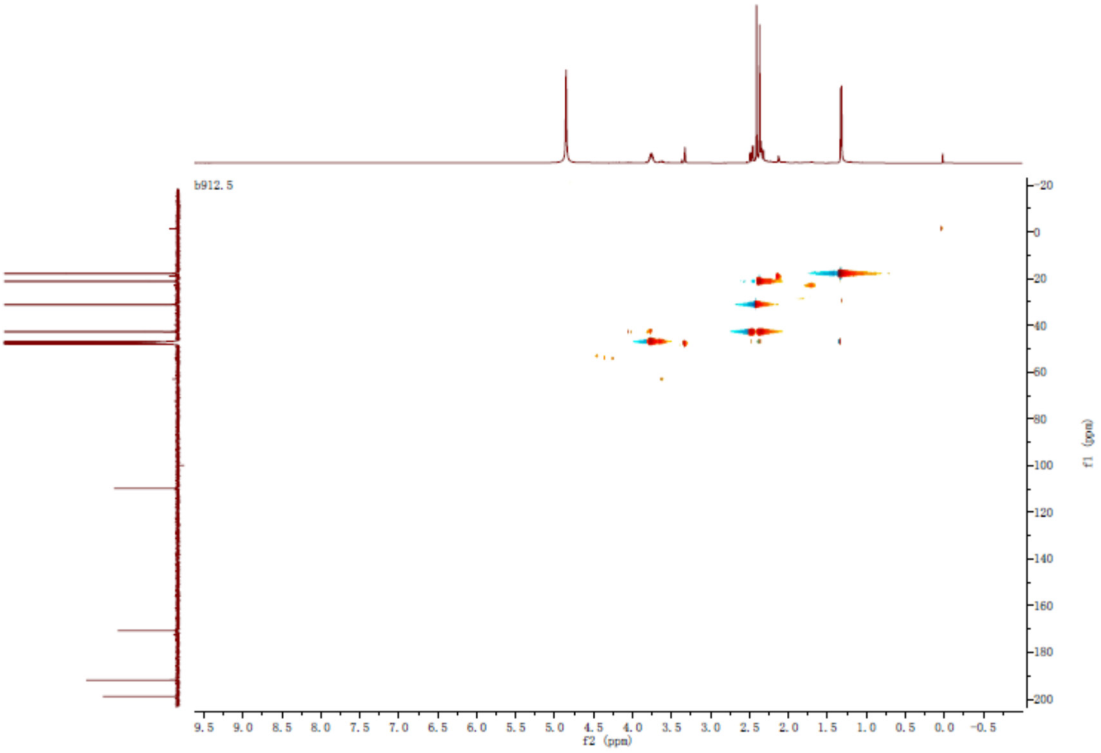

Figure S3. HSQC spectrum of compound 2（500 MHz, CD_3OD）

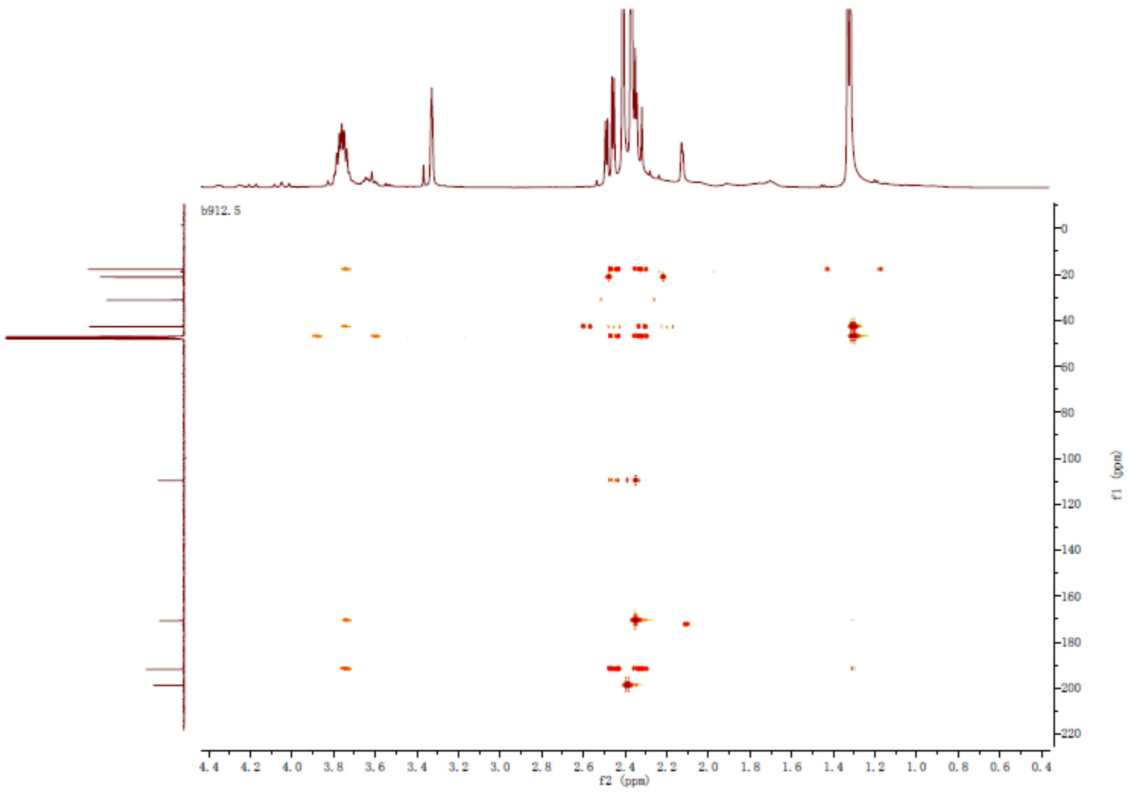

Figure S4. HMBC spectrum of compound 2（500 MHz, CD_3OD）

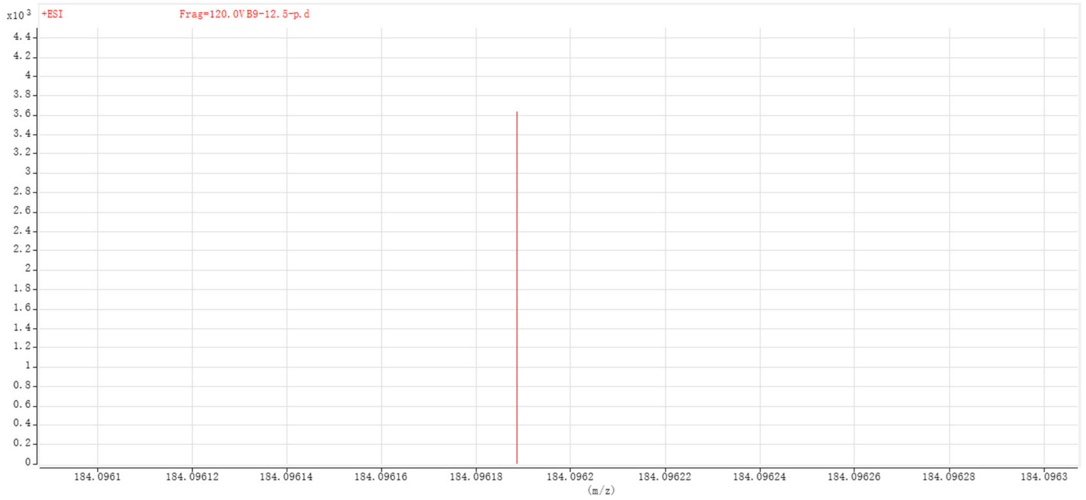

Figure S5. HR-ESI-MS spectrum of compound 2

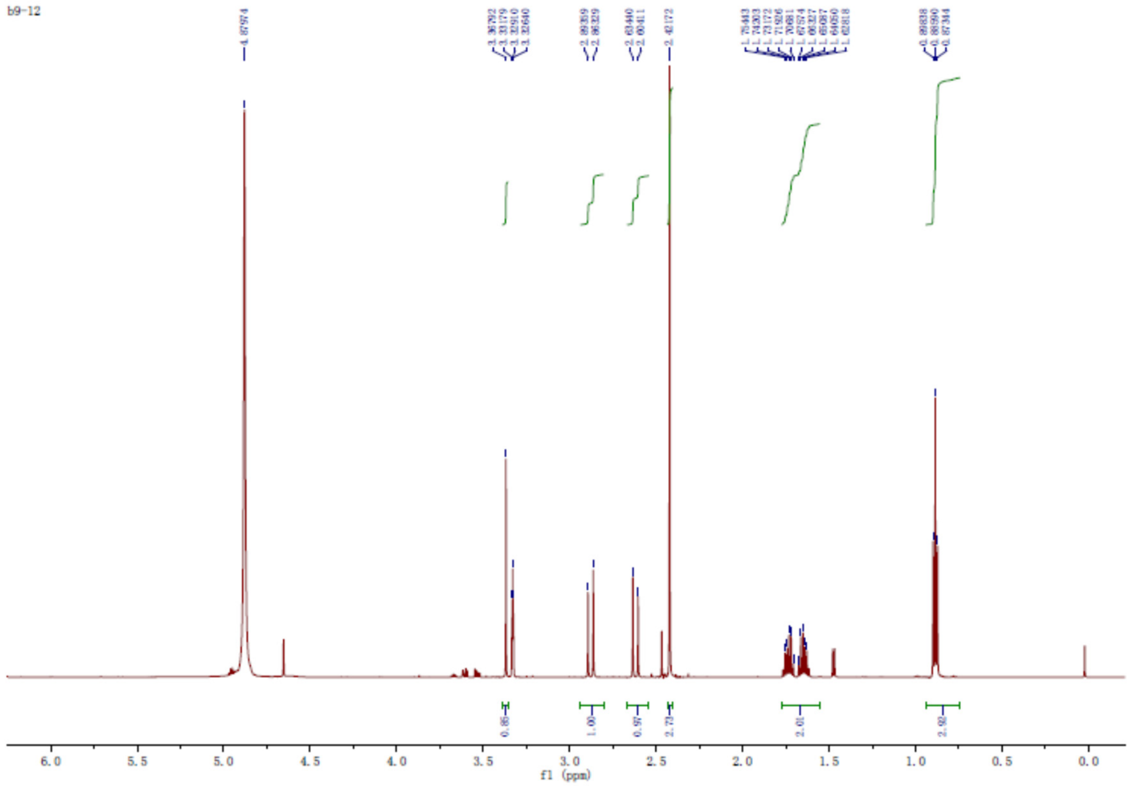

Figure S6. ^{1}H NMR spectrum of compound 3（500 MHz, CD_3OD）

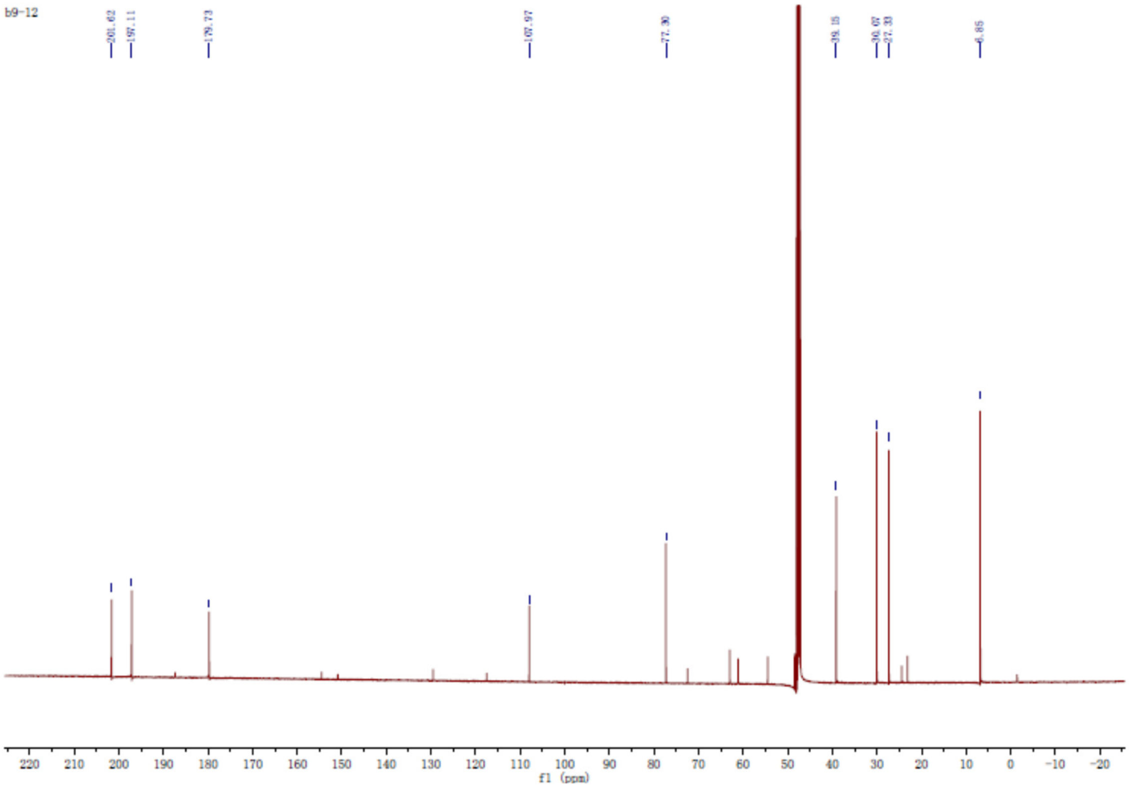

Figure S7. ^{13}C NMR spectrum of compound 3（125 MHz, CD_3OD）

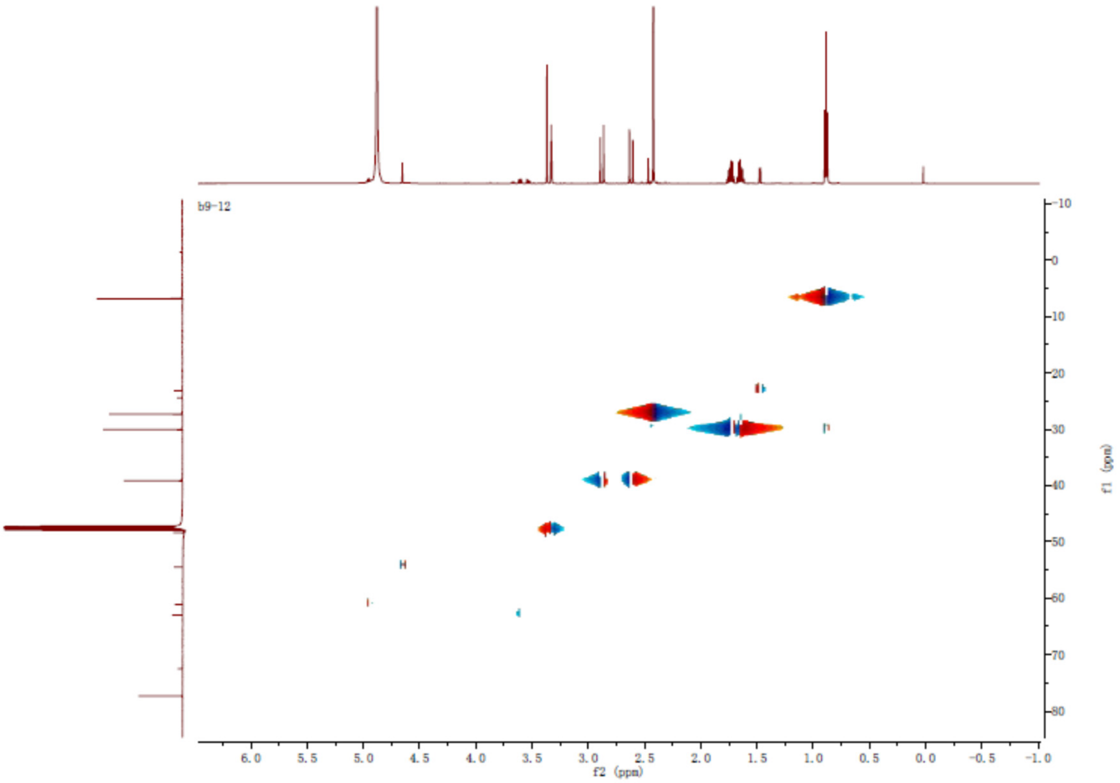

Figure S8. HSQC spectrum of compound 3（500 MHz, CD_3OD）

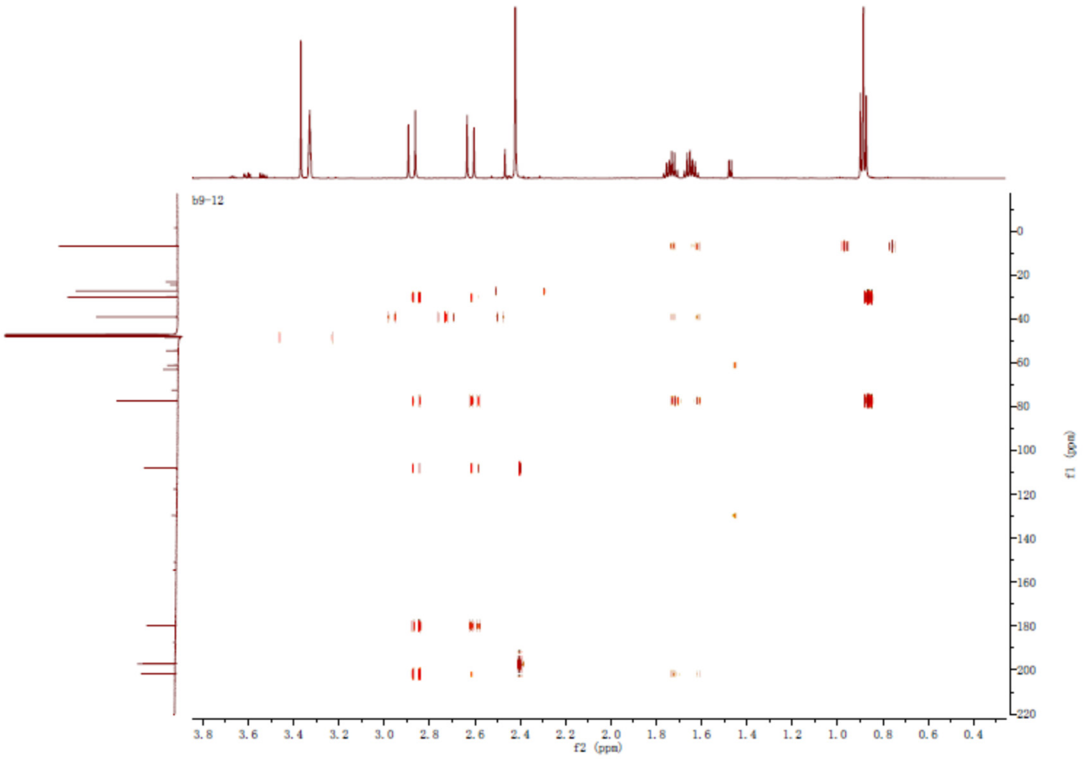

Figure S9. HMBC spectrum of compound 3（500 MHz, CD_3OD）

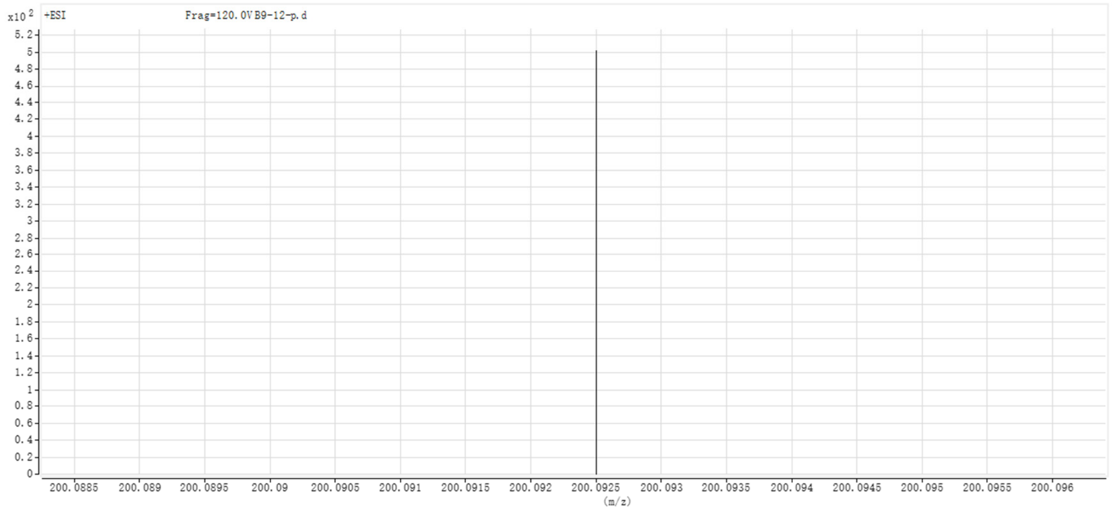

Figure S10. HR-ESI-MS spectrum of compound 3

Table S1. Gibbs free energies[a] and equilibrium populations[b] of low-energy conformers of 2S.

**Conformers**	**ΔG(a.u.)**	**P(%)/100**	**G(a.u.)**
**2S000001_tddft_**	0.00026	30.53	-631.097595
**2S000002_tddft_**	0.0003	29.29	-631.097556
**2S000003_tddft_**	0.0000	40.19	-631.097855

[a]wB97M-V/def2-TZVP, in a.u.

[b]From ΔG values at 298.15K.

Table S2. Cartesian coordinates for the low-energy reoptimized random reseach conformers of 2S at B3LYP-D3(BJ)/6-31G* level of theory in methanol.

**2S000001_en_**		**Standard Orientation (A.U.)**			
**Center number**	Atomic number	Atomic Type	X	Y	Z
**0**	6	0	-1.252163	2.390848	-1.210697
**1**	6	0	-0.551021	1.737358	1.236701
**2**	6	0	-0.067711	-1.925404	-2.643984
**3**	6	0	-0.427787	0.832291	-3.409391
**4**	8	0	-2.594725	4.388984	-1.712095
**5**	6	0	-2.591174	-3.295054	-2.266344
**6**	7	0	1.53058	-2.031793	-0.383603
**7**	6	0	1.072105	-0.498603	1.675054
**8**	8	0	2.030331	-0.960287	3.744403
**9**	6	0	2.922498	-4.36677	0.070769
**10**	6	0	-0.824819	2.875249	5.988391
**11**	6	0	-1.396519	3.362513	3.264935
**12**	8	0	-2.70314	5.288146	2.733483
**13**	1	0	0.98157	-2.876155	-4.160421
**14**	1	0	-1.813373	1.017907	-4.933938
**15**	1	0	1.367717	1.619745	-4.098481
**16**	1	0	-2.898878	5.194811	0.129703
**17**	1	0	-3.668876	-3.334878	-4.032454
**18**	1	0	-2.280101	-5.246953	-1.661183
**19**	1	0	-3.733091	-2.344755	-0.825239
**20**	1	0	3.712336	-5.038336	-1.718906
**21**	1	0	4.451627	-3.998346	1.403831
**22**	1	0	1.724103	-5.858581	0.881686
**23**	1	0	-1.329961	0.943085	6.515643
**24**	1	0	1.210657	3.01567	6.319913
**25**	1	0	-1.847668	4.255491	7.130119

2S000002_en_		Standard Orientation (A.U.)			
**Center number**	Atomic number	Atomic Type	X	Y	Z
**0**	6	0	-2.264766	2.006203	-0.62382
**1**	6	0	-1.158164	1.225066	1.628925
**2**	6	0	0.205513	-1.273008	-2.911047
**3**	6	0	-1.156077	1.262675	-3.105867
**4**	8	0	-4.256476	3.447025	-0.687765
**5**	6	0	-1.640741	-3.498515	-2.758717
**6**	7	0	1.940457	-1.201567	-0.752781
**7**	6	0	1.174381	-0.318702	1.577924
**8**	8	0	2.422829	-0.757252	3.491779
**9**	6	0	4.097014	-2.916331	-0.808206
**10**	6	0	-1.371864	1.326761	6.516496
**11**	6	0	-2.324072	2.053549	3.957762
**12**	8	0	-4.259481	3.446363	3.848981
**13**	1	0	1.37643	-1.499547	-4.608882
**14**	1	0	-2.651875	1.208497	-4.533378
**15**	1	0	0.185029	2.749521	-3.661806
**16**	1	0	-4.64825	3.751406	1.284632
**17**	1	0	-2.793959	-3.605651	-4.473655
**18**	1	0	-0.618908	-5.284531	-2.561746
**19**	1	0	-2.905561	-3.294356	-1.132933
**20**	1	0	3.56935	-4.8669	-0.322343
**21**	1	0	4.923614	-2.897328	-2.704241
**22**	1	0	5.500382	-2.284785	0.563449
**23**	1	0	0.512628	2.119448	6.82186
**24**	1	0	-2.69724	2.027735	7.933087
**25**	1	0	-1.131354	-0.71967	6.661781

**2S000003_en_**		**Standard Orientation (A.U.)**			
**Center number**	Atomic number	Atomic Type	X	Y	Z
**0**	6	0	-1.851894	2.151947	-1.155686
**1**	6	0	-0.840753	1.518204	1.276456
**2**	6	0	-0.558165	-2.17153	-2.592124
**3**	6	0	-1.074602	0.555397	-3.37177
**4**	8	0	-3.300306	4.000032	-1.506667
**5**	6	0	-2.992791	-3.610997	-1.969427
**6**	7	0	1.234126	-2.187666	-0.479237
**7**	6	0	0.923433	-0.632117	1.584426
**8**	8	0	2.102595	-1.007062	3.557304
**9**	6	0	2.772702	-4.448931	-0.146001
**10**	6	0	-0.706712	2.678296	6.005926
**11**	6	0	-1.529582	3.029615	3.340454
**12**	8	0	-3.050161	4.948915	2.993293
**13**	1	0	0.387134	-3.123659	-4.175102
**14**	1	0	-2.543454	0.667536	-4.822107
**15**	1	0	0.654555	1.398368	-4.156892
**16**	1	0	-3.454517	4.965055	1.061306
**17**	1	0	-4.223415	-3.710047	-3.62987
**18**	1	0	-2.569842	-5.542581	-1.36649
**19**	1	0	-4.031325	-2.669984	-0.445567
**20**	1	0	4.358994	-4.013724	1.096586
**21**	1	0	1.70282	-6.009742	0.713638
**22**	1	0	3.482431	-5.061003	-1.990055
**23**	1	0	1.32431	3.027992	6.168387
**24**	1	0	-1.759121	3.984139	7.207529
**25**	1	0	-0.973309	0.722295	6.607932

Table S3. Compound 3 NMR Boltzmann averaged isotropic magnetic shielding values (σ), unscaled (δu) and scaled (δs) chemical shifts calculated at the B3LYP/6-31G**//MMFF level of theory for conformers of **6R** and **6S**.

No.	δexp	6R		6S	
		δu	δs	δu	δs
2	179.7	172.5	168.8	172.7	168.7
3	107.9	110.1	105.6	110.1	105.4
4	201.6	203.7	200.3	199.9	196.2
5	39.2	45.7	40.5	44.3	39.0
6	77.3	86.3	81.6	86.5	81.6
7	197.1	209.5	206.2	213.7	210.1
8	27.3	31.2	25.9	33.2	27.8
9	30.1	38.5	33.2	38.4	33
10	6.85	10.6	5.0	11.0	5.3

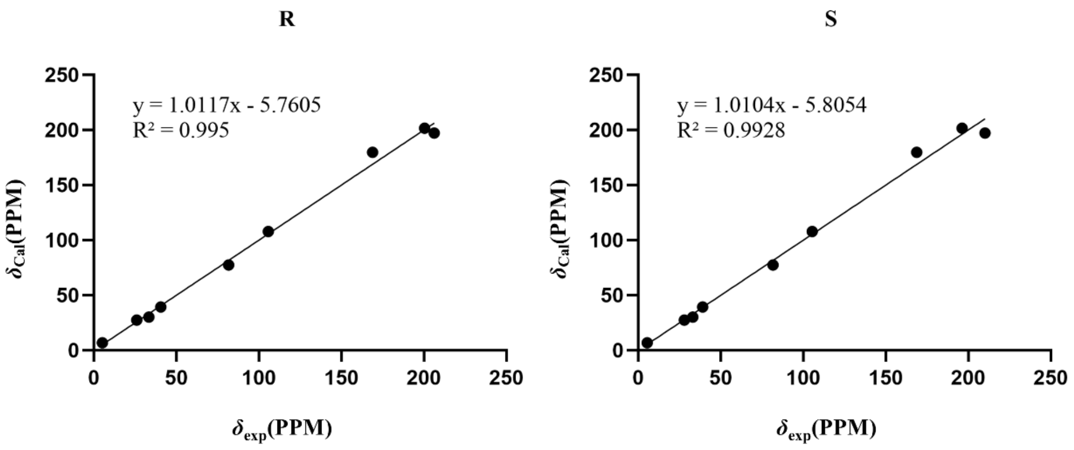

Figure S11. The similar chemical shifts between experiment and scaled chemical shift of compound 3 (6R and 6S, respectively). The linear fitting is shown as dashed line

Table S4. Compound 3 Gibbs free energies[a] and equilibrium populations[b] of low-energy conformers of YST-MN-4b.

**Conformers**	**ΔG**	**P(%)/100**
**YST-MN-4b000001_tddft_**	0.00041	31.46
**YST-MN-4b000002_tddft_**	0.0	48.47
**YST-MN-4b000003_tddft_**	0.0009	18.72
**YST-MN-4b000004_tddft_**	0.00338	1.35

[a]wB97M-V/def2-TZVP, in kcal/mol.

[b]From ΔG values at 298.15K.

Table S5 Compound 3 cartesian coordinates for the low-energy reoptimized random reseach conformers of YST-MN-4b at B3LYP-D3(BJ)/6-31G** level of theory in methanol.

**YST-MN-4b000001_en_**		**Standard Orientation (Ångstroms)**			
**Center number**	Atomic number	Atomic Type	X	Y	Z
**0**	6	0	7.211499	3.61842	4.213476
**1**	6	0	6.177522	1.445937	2.626178
**2**	6	0	5.336601	-0.766312	4.26931
**3**	6	0	3.455554	0.099622	6.28239
**4**	6	0	4.492004	2.247407	7.861954
**5**	6	0	5.438264	4.496011	6.298779
**6**	8	0	1.236609	1.065723	5.021354
**7**	6	0	-0.552354	-0.623862	4.265132
**8**	6	0	-2.362574	0.629265	2.591992
**9**	6	0	-4.124891	-0.722114	1.379795
**10**	6	0	-5.83468	0.25064	-0.530852
**11**	6	0	-6.422447	-1.172237	-2.693349
**12**	6	0	-6.813459	2.702059	-0.332212
**13**	6	0	-8.339633	3.701195	-2.187378
**14**	6	0	-8.924319	2.271515	-4.342085
**15**	6	0	-7.947862	-0.143302	-4.569863
**16**	8	0	-0.542437	-2.83949	4.858477
**17**	6	0	3.247128	6.05105	5.212927
**18**	8	0	6.889069	6.16529	7.862456
**19**	8	0	2.857708	6.52392	3.020692
**20**	8	0	1.792656	6.994392	7.113633
**21**	8	0	8.040525	0.619438	0.855337
**22**	8	0	7.466901	-2.001893	5.383853
**23**	8	0	-10.422264	3.217834	-6.185716
**24**	8	0	-9.399943	6.059793	-2.111712
**25**	6	0	-5.281593	-3.7508	-3.172148
**26**	6	0	-7.394277	-5.694436	-3.300318
**27**	8	0	-8.415407	-6.463511	-5.180954
**28**	8	0	-8.05832	-6.396326	-0.928065
**29**	8	0	-3.935997	-3.866261	-5.499576
**30**	6	0	2.490671	-4.820372	-3.541981
**31**	6	0	3.399585	-2.150029	-2.949351
**32**	6	0	2.09737	-0.201038	-4.603978
**33**	6	0	-0.737051	-0.60432	-4.632827
**34**	6	0	-1.341598	-3.32745	-5.350555
**35**	8	0	-0.213081	-4.974144	-3.56851
**36**	6	0	3.428333	-6.726958	-1.622698
**37**	8	0	2.706957	2.225293	-3.623477
**38**	8	0	6.044307	-2.120386	-3.284946

**39**	8	0	-1.77702	1.163969	-6.364121
**40**	8	0	2.897457	-6.066515	0.9225
**41**	1	0	7.68231	5.191242	2.981612
**42**	1	0	8.968948	3.037527	5.127331
**43**	1	0	4.585994	2.112168	1.511926
**44**	1	0	4.460518	-2.219491	3.109894
**45**	1	0	2.933359	-1.506659	7.453388
**46**	1	0	3.081281	2.854971	9.229501
**47**	1	0	6.104463	1.564912	8.951302
**48**	1	0	-2.047381	2.61091	2.204465
**49**	1	0	-4.188507	-2.72283	1.801336
**50**	1	0	-6.402453	3.811244	1.338107
**51**	1	0	-8.377343	-1.226124	-6.248357
**52**	1	0	5.769417	6.867464	9.121358
**53**	1	0	0.536828	8.064878	6.31758
**54**	1	0	9.05958	-0.621546	1.736499
**55**	1	0	8.29033	-0.838682	6.521392
**56**	1	0	-10.895751	4.921708	-5.724151
**57**	1	0	-8.845283	6.944611	-0.620617
**58**	1	0	-4.038271	-4.287067	-1.638196
**59**	1	0	-9.479287	-7.542583	-1.104466
**60**	1	0	3.224942	-5.371226	-5.402852
**61**	1	0	2.897048	-1.737482	-0.989778
**62**	1	0	2.819801	-0.396466	-6.537241
**63**	1	0	-1.434247	-0.260434	-2.723699
**64**	1	0	-0.637737	-3.724083	-7.25553
**65**	1	0	2.645276	-8.582173	-2.093253
**66**	1	0	5.473395	-6.827265	-1.771038
**67**	1	0	1.791267	3.443876	-4.623858
**68**	1	0	6.763503	-0.9846	-2.026618
**69**	1	0	-3.595008	1.030588	-6.260738
**70**	1	0	1.090774	-6.027189	1.157721

YST-MN-4b000001_tddft_		Standard Orientation (Ångstroms)			
**Center number**	Atomic number	Atomic Type	X	Y	Z
**0**	6	0	7.211499	3.61842	4.213476
**1**	6	0	6.177522	1.445937	2.626178
**2**	6	0	5.336601	-0.766312	4.26931
**3**	6	0	3.455554	0.099622	6.28239
**4**	6	0	4.492004	2.247407	7.861954
**5**	6	0	5.438264	4.496011	6.298779
**6**	8	0	1.236609	1.065723	5.021354
**7**	6	0	-0.552354	-0.623862	4.265132
**8**	6	0	-2.362574	0.629265	2.591992
**9**	6	0	-4.124891	-0.722114	1.379795
**10**	6	0	-5.83468	0.25064	-0.530852
**11**	6	0	-6.422447	-1.172237	-2.693349
**12**	6	0	-6.813459	2.702059	-0.332212
**13**	6	0	-8.339633	3.701195	-2.187378
**14**	6	0	-8.924319	2.271515	-4.342085
**15**	6	0	-7.947862	-0.143302	-4.569863
**16**	8	0	-0.542437	-2.83949	4.858477
**17**	6	0	3.247128	6.05105	5.212927
**18**	8	0	6.889069	6.16529	7.862456
**19**	8	0	2.857708	6.52392	3.020692
**20**	8	0	1.792656	6.994392	7.113633
**21**	8	0	8.040525	0.619438	0.855337
**22**	8	0	7.466901	-2.001893	5.383853
**23**	8	0	-10.422264	3.217834	-6.185716
**24**	8	0	-9.399943	6.059793	-2.111712
**25**	6	0	-5.281593	-3.7508	-3.172148
**26**	6	0	-7.394277	-5.694436	-3.300318
**27**	8	0	-8.415407	-6.463511	-5.180954
**28**	8	0	-8.05832	-6.396326	-0.928065
**29**	8	0	-3.935997	-3.866261	-5.499576
**30**	6	0	2.490671	-4.820372	-3.541981
**31**	6	0	3.399585	-2.150029	-2.949351
**32**	6	0	2.09737	-0.201038	-4.603978
**33**	6	0	-0.737051	-0.60432	-4.632827
**34**	6	0	-1.341598	-3.32745	-5.350555
**35**	8	0	-0.213081	-4.974144	-3.56851
**36**	6	0	3.428333	-6.726958	-1.622698
**37**	8	0	2.706957	2.225293	-3.623477
**38**	8	0	6.044307	-2.120386	-3.284946
**39**	8	0	-1.77702	1.163969	-6.364121
**40**	8	0	2.897457	-6.066515	0.9225

**41**	1	0	7.68231	5.191242	2.981612
**42**	1	0	8.968948	3.037527	5.127331
**43**	1	0	4.585994	2.112168	1.511926
**44**	1	0	4.460518	-2.219491	3.109894
**45**	1	0	2.933359	-1.506659	7.453388
**46**	1	0	3.081281	2.854971	9.229501
**47**	1	0	6.104463	1.564912	8.951302
**48**	1	0	-2.047381	2.61091	2.204465
**49**	1	0	-4.188507	-2.72283	1.801336
**50**	1	0	-6.402453	3.811244	1.338107
**51**	1	0	-8.377343	-1.226124	-6.248357
**52**	1	0	5.769417	6.867464	9.121358
**53**	1	0	0.536828	8.064878	6.31758
**54**	1	0	9.05958	-0.621546	1.736499
**55**	1	0	8.29033	-0.838682	6.521392
**56**	1	0	-10.895751	4.921708	-5.724151
**57**	1	0	-8.845283	6.944611	-0.620617
**58**	1	0	-4.038271	-4.287067	-1.638196
**59**	1	0	-9.479287	-7.542583	-1.104466
**60**	1	0	3.224942	-5.371226	-5.402852
**61**	1	0	2.897048	-1.737482	-0.989778
**62**	1	0	2.819801	-0.396466	-6.537241
**63**	1	0	-1.434247	-0.260434	-2.723699
**64**	1	0	-0.637737	-3.724083	-7.25553
**65**	1	0	2.645276	-8.582173	-2.093253
**66**	1	0	5.473395	-6.827265	-1.771038
**67**	1	0	1.791267	3.443876	-4.623858
**68**	1	0	6.763503	-0.9846	-2.026618
**69**	1	0	-3.595008	1.030588	-6.260738
**70**	1	0	1.090774	-6.027189	1.157721

YST-MN-4b000002_en_		Standard Orientation (Ångstroms)			
**Center number**	Atomic number	Atomic Type	X	Y	Z
**0**	6	0	8.303589	0.013666	5.091387
**1**	6	0	5.871897	-0.862377	6.363432
**2**	6	0	4.354042	1.363373	7.36599
**3**	6	0	3.922161	3.387136	5.347381
**4**	6	0	6.341803	4.171814	4.044107
**5**	6	0	7.881912	1.96504	3.02071
**6**	8	0	2.186056	2.439933	3.44951
**7**	6	0	-0.309774	2.472984	4.073831
**8**	6	0	-1.855388	1.47022	2.006758
**9**	6	0	-4.298318	0.953739	2.416835
**10**	6	0	-6.181434	-0.062468	0.703031
**11**	6	0	-6.431634	0.49155	-1.886126
**12**	6	0	-7.960481	-1.687352	1.807117
**13**	6	0	-9.849409	-2.818298	0.42156
**14**	6	0	-10.09977	-2.251524	-2.146401
**15**	6	0	-8.410645	-0.581799	-3.236942
**16**	8	0	-1.094104	3.240284	6.093256
**17**	6	0	6.631876	0.776239	0.681282
**18**	8	0	10.239619	3.027842	2.207163
**19**	8	0	6.333785	-1.504564	0.455777
**20**	8	0	6.027176	2.458816	-1.057984
**21**	8	0	6.35901	-2.628941	8.319832
**22**	8	0	5.76108	2.435204	9.41967
**23**	8	0	-11.966561	-3.281582	-3.562081
**24**	8	0	-11.597012	-4.468655	1.371812
**25**	6	0	-4.685842	2.187682	-3.363175
**26**	6	0	-4.144682	4.716726	-2.10264
**27**	8	0	-5.752623	5.949906	-1.064431
**28**	8	0	-1.745853	5.524124	-2.447288
**29**	8	0	-2.372737	1.020388	-4.1131
**30**	6	0	-0.87941	-4.725918	-3.655089
**31**	6	0	1.436255	-3.064248	-3.234642
**32**	6	0	2.04315	-1.367469	-5.521509
**33**	6	0	-0.193953	-1.089044	-7.338302
**34**	6	0	-2.628534	-1.072519	-5.820373
**35**	8	0	-3.049176	-3.328019	-4.565602
**36**	6	0	-1.744684	-5.953765	-1.21436
**37**	8	0	2.91275	1.092235	-4.760718
**38**	8	0	3.467769	-4.680815	-2.632571
**39**	8	0	-0.391943	-3.132306	-9.058876
**40**	8	0	-3.989812	-7.38045	-1.528301

**41**	1	0	9.272733	-1.636174	4.335513
**42**	1	0	9.524688	0.889029	6.500232
**43**	1	0	4.712478	-1.856644	4.987642
**44**	1	0	2.535723	0.674685	8.04132
**45**	1	0	3.047897	5.020432	6.244385
**46**	1	0	5.943727	5.518175	2.546098
**47**	1	0	7.518996	5.118261	5.440899
**48**	1	0	-0.936796	1.098756	0.229825
**49**	1	0	-4.955822	1.260379	4.335607
**50**	1	0	-7.814111	-2.092458	3.808781
**51**	1	0	-8.673268	-0.141234	-5.214416
**52**	1	0	11.360779	1.643311	1.812891
**53**	1	0	5.086894	1.639862	-2.471161
**54**	1	0	6.991991	-1.656886	9.731424
**55**	1	0	4.580674	3.215708	10.561857
**56**	1	0	-12.934275	-4.406558	-2.494704
**57**	1	0	-11.155573	-4.934388	3.075648
**58**	1	0	-5.703594	2.734874	-5.083421
**59**	1	0	-1.656679	7.177957	-1.65778
**60**	1	0	-0.437094	-6.172724	-5.054059
**61**	1	0	1.027183	-1.807554	-1.645414
**62**	1	0	3.618352	-2.230454	-6.525672
**63**	1	0	-0.019726	0.716598	-8.329644
**64**	1	0	-4.262351	-0.767429	-7.039274
**65**	1	0	-0.287625	-7.240774	-0.556918
**66**	1	0	-2.001295	-4.47421	0.215343
**67**	1	0	1.434059	2.001496	-4.165929
**68**	1	0	4.653565	-3.696354	-1.622217
**69**	1	0	1.092114	-3.125648	-10.11456
**70**	1	0	-5.191129	-6.269238	-2.336187

YST-MN-4b000002_tddft_		Standard Orientation (Ångstroms)			
**Center number**	Atomic number	Atomic Type	X	Y	Z
**0**	6	0	8.303589	0.013666	5.091387
**1**	6	0	5.871897	-0.862377	6.363432
**2**	6	0	4.354042	1.363373	7.36599
**3**	6	0	3.922161	3.387136	5.347381
**4**	6	0	6.341803	4.171814	4.044107
**5**	6	0	7.881912	1.96504	3.02071
**6**	8	0	2.186056	2.439933	3.44951
**7**	6	0	-0.309774	2.472984	4.073831
**8**	6	0	-1.855388	1.47022	2.006758
**9**	6	0	-4.298318	0.953739	2.416835
**10**	6	0	-6.181434	-0.062468	0.703031
**11**	6	0	-6.431634	0.49155	-1.886126
**12**	6	0	-7.960481	-1.687352	1.807117
**13**	6	0	-9.849409	-2.818298	0.42156
**14**	6	0	-10.09977	-2.251524	-2.146401
**15**	6	0	-8.410645	-0.581799	-3.236942
**16**	8	0	-1.094104	3.240284	6.093256
**17**	6	0	6.631876	0.776239	0.681282
**18**	8	0	10.239619	3.027842	2.207163
**19**	8	0	6.333785	-1.504564	0.455777
**20**	8	0	6.027176	2.458816	-1.057984
**21**	8	0	6.35901	-2.628941	8.319832
**22**	8	0	5.76108	2.435204	9.41967
**23**	8	0	-11.966561	-3.281582	-3.562081
**24**	8	0	-11.597012	-4.468655	1.371812
**25**	6	0	-4.685842	2.187682	-3.363175
**26**	6	0	-4.144682	4.716726	-2.10264
**27**	8	0	-5.752623	5.949906	-1.064431
**28**	8	0	-1.745853	5.524124	-2.447288
**29**	8	0	-2.372737	1.020388	-4.1131
**30**	6	0	-0.87941	-4.725918	-3.655089
**31**	6	0	1.436255	-3.064248	-3.234642
**32**	6	0	2.04315	-1.367469	-5.521509
**33**	6	0	-0.193953	-1.089044	-7.338302
**34**	6	0	-2.628534	-1.072519	-5.820373
**35**	8	0	-3.049176	-3.328019	-4.565602
**36**	6	0	-1.744684	-5.953765	-1.21436
**37**	8	0	2.91275	1.092235	-4.760718
**38**	8	0	3.467769	-4.680815	-2.632571
**39**	8	0	-0.391943	-3.132306	-9.058876
**40**	8	0	-3.989812	-7.38045	-1.528301

**41**	1	0	9.272733	-1.636174	4.335513
**42**	1	0	9.524688	0.889029	6.500232
**43**	1	0	4.712478	-1.856644	4.987642
**44**	1	0	2.535723	0.674685	8.04132
**45**	1	0	3.047897	5.020432	6.244385
**46**	1	0	5.943727	5.518175	2.546098
**47**	1	0	7.518996	5.118261	5.440899
**48**	1	0	-0.936796	1.098756	0.229825
**49**	1	0	-4.955822	1.260379	4.335607
**50**	1	0	-7.814111	-2.092458	3.808781
**51**	1	0	-8.673268	-0.141234	-5.214416
**52**	1	0	11.360779	1.643311	1.812891
**53**	1	0	5.086894	1.639862	-2.471161
**54**	1	0	6.991991	-1.656886	9.731424
**55**	1	0	4.580674	3.215708	10.561857
**56**	1	0	-12.934275	-4.406558	-2.494704
**57**	1	0	-11.155573	-4.934388	3.075648
**58**	1	0	-5.703594	2.734874	-5.083421
**59**	1	0	-1.656679	7.177957	-1.65778
**60**	1	0	-0.437094	-6.172724	-5.054059
**61**	1	0	1.027183	-1.807554	-1.645414
**62**	1	0	3.618352	-2.230454	-6.525672
**63**	1	0	-0.019726	0.716598	-8.329644
**64**	1	0	-4.262351	-0.767429	-7.039274
**65**	1	0	-0.287625	-7.240774	-0.556918
**66**	1	0	-2.001295	-4.47421	0.215343
**67**	1	0	1.434059	2.001496	-4.165929
**68**	1	0	4.653565	-3.696354	-1.622217
**69**	1	0	1.092114	-3.125648	-10.11456
**70**	1	0	-5.191129	-6.269238	-2.336187

**YST-MN-4b000003_en_**		**Standard Orientation (Ångstroms)**			
**Center number**	Atomic number	Atomic Type	X	Y	Z
**0**	6	0	8.132917	0.099861	5.164026
**1**	6	0	5.685165	-0.702108	6.458337
**2**	6	0	4.189169	1.563707	7.401795
**3**	6	0	3.770526	3.533425	5.32921
**4**	6	0	6.202053	4.261255	4.015402
**5**	6	0	7.764453	2.022631	3.055905
**6**	8	0	2.039654	2.526238	3.461784
**7**	6	0	-0.460713	2.655292	4.049161
**8**	6	0	-1.997598	1.477832	2.067757
**9**	6	0	-4.483885	1.201626	2.42541
**10**	6	0	-6.333198	0.034206	0.764732
**11**	6	0	-6.451751	0.297727	-1.878375
**12**	6	0	-8.187765	-1.435649	1.956523
**13**	6	0	-10.01958	-2.702379	0.611061
**14**	6	0	-10.114002	-2.461432	-2.019378
**15**	6	0	-8.353912	-0.938818	-3.206387
**16**	8	0	-1.255919	3.620055	5.977454
**17**	6	0	6.533581	0.807899	0.72367
**18**	8	0	10.150448	2.910728	2.121314
**19**	8	0	5.994711	-1.432359	0.622023
**20**	8	0	6.156899	2.454226	-1.107848
**21**	8	0	6.148711	-2.424077	8.460794
**22**	8	0	5.609328	2.68026	9.424648
**23**	8	0	-11.911319	-3.64783	-3.402859
**24**	8	0	-11.840898	-4.210081	1.654307
**25**	6	0	-4.668896	1.892254	-3.420641
**26**	6	0	-4.127307	4.484428	-2.293314
**27**	8	0	-5.750879	5.806882	-1.399333
**28**	8	0	-1.703048	5.229998	-2.594071
**29**	8	0	-2.360614	0.656684	-4.054479
**30**	6	0	-0.455555	-4.863864	-4.202811
**31**	6	0	1.684817	-3.02967	-3.597958
**32**	6	0	2.203239	-1.15979	-5.761591
**33**	6	0	-0.052665	-0.838789	-7.555587
**34**	6	0	-2.472092	-1.150654	-6.052265
**35**	8	0	-2.719462	-3.634383	-5.175463
**36**	6	0	-1.308483	-6.37719	-1.924755
**37**	8	0	2.978447	1.265581	-4.806428
**38**	8	0	3.829923	-4.509476	-3.042988
**39**	8	0	0.002929	-2.543318	-9.619885
**40**	8	0	-2.325924	-4.841003	0.012679

**41**	1	0	9.08663	-1.567573	4.439335
**42**	1	0	9.357087	0.96536	6.587075
**43**	1	0	4.519707	-1.714481	5.102534
**44**	1	0	2.365757	0.907379	8.099517
**45**	1	0	2.897806	5.194754	6.174583
**46**	1	0	5.828673	5.566822	2.475312
**47**	1	0	7.358014	5.252791	5.402574
**48**	1	0	-1.03827	0.767671	0.418833
**49**	1	0	-5.208702	1.83824	4.235407
**50**	1	0	-8.139772	-1.614449	3.995556
**51**	1	0	-8.513112	-0.720154	-5.231262
**52**	1	0	11.254721	3.096846	3.559358
**53**	1	0	5.187118	1.677728	-2.527454
**54**	1	0	6.780518	-1.425076	9.85365
**55**	1	0	4.441023	3.529016	10.530168
**56**	1	0	-12.968852	-4.60778	-2.262657
**57**	1	0	-11.594674	-4.332751	3.454137
**58**	1	0	-5.651983	2.359391	-5.183006
**59**	1	0	-1.610388	6.928857	-1.907422
**60**	1	0	0.226708	-6.197403	-5.631061
**61**	1	0	1.117881	-1.941103	-1.940398
**62**	1	0	3.786989	-1.869547	-6.85817
**63**	1	0	0.023609	1.053676	-8.361192
**64**	1	0	-4.138363	-0.808564	-7.215548
**65**	1	0	-2.680369	-7.795841	-2.550587
**66**	1	0	0.318912	-7.35202	-1.1388
**67**	1	0	1.484728	2.045682	-4.085076
**68**	1	0	4.793524	-3.585975	-1.771731
**69**	1	0	-0.748885	-4.114729	-9.081296
**70**	1	0	-3.712451	-3.909245	-0.72463

YST-MN-4b000003_tddft_		Standard Orientation (Ångstroms)			
**Center number**	Atomic number	Atomic Type	X	Y	Z
**0**	6	0	8.132917	0.099861	5.164026
**1**	6	0	5.685165	-0.702108	6.458337
**2**	6	0	4.189169	1.563707	7.401795
**3**	6	0	3.770526	3.533425	5.32921
**4**	6	0	6.202053	4.261255	4.015402
**5**	6	0	7.764453	2.022631	3.055905
**6**	8	0	2.039654	2.526238	3.461784
**7**	6	0	-0.460713	2.655292	4.049161
**8**	6	0	-1.997598	1.477832	2.067757
**9**	6	0	-4.483885	1.201626	2.42541
**10**	6	0	-6.333198	0.034206	0.764732
**11**	6	0	-6.451751	0.297727	-1.878375
**12**	6	0	-8.187765	-1.435649	1.956523
**13**	6	0	-10.01958	-2.702379	0.611061
**14**	6	0	-10.114002	-2.461432	-2.019378
**15**	6	0	-8.353912	-0.938818	-3.206387
**16**	8	0	-1.255919	3.620055	5.977454
**17**	6	0	6.533581	0.807899	0.72367
**18**	8	0	10.150448	2.910728	2.121314
**19**	8	0	5.994711	-1.432359	0.622023
**20**	8	0	6.156899	2.454226	-1.107848
**21**	8	0	6.148711	-2.424077	8.460794
**22**	8	0	5.609328	2.68026	9.424648
**23**	8	0	-11.911319	-3.64783	-3.402859
**24**	8	0	-11.840898	-4.210081	1.654307
**25**	6	0	-4.668896	1.892254	-3.420641
**26**	6	0	-4.127307	4.484428	-2.293314
**27**	8	0	-5.750879	5.806882	-1.399333
**28**	8	0	-1.703048	5.229998	-2.594071
**29**	8	0	-2.360614	0.656684	-4.054479
**30**	6	0	-0.455555	-4.863864	-4.202811
**31**	6	0	1.684817	-3.02967	-3.597958
**32**	6	0	2.203239	-1.15979	-5.761591
**33**	6	0	-0.052665	-0.838789	-7.555587
**34**	6	0	-2.472092	-1.150654	-6.052265
**35**	8	0	-2.719462	-3.634383	-5.175463
**36**	6	0	-1.308483	-6.37719	-1.924755
**37**	8	0	2.978447	1.265581	-4.806428
**38**	8	0	3.829923	-4.509476	-3.042988
**39**	8	0	0.002929	-2.543318	-9.619885
**40**	8	0	-2.325924	-4.841003	0.012679

**41**	1	0	9.08663	-1.567573	4.439335
**42**	1	0	9.357087	0.96536	6.587075
**43**	1	0	4.519707	-1.714481	5.102534
**44**	1	0	2.365757	0.907379	8.099517
**45**	1	0	2.897806	5.194754	6.174583
**46**	1	0	5.828673	5.566822	2.475312
**47**	1	0	7.358014	5.252791	5.402574
**48**	1	0	-1.03827	0.767671	0.418833
**49**	1	0	-5.208702	1.83824	4.235407
**50**	1	0	-8.139772	-1.614449	3.995556
**51**	1	0	-8.513112	-0.720154	-5.231262
**52**	1	0	11.254721	3.096846	3.559358
**53**	1	0	5.187118	1.677728	-2.527454
**54**	1	0	6.780518	-1.425076	9.85365
**55**	1	0	4.441023	3.529016	10.530168
**56**	1	0	-12.968852	-4.60778	-2.262657
**57**	1	0	-11.594674	-4.332751	3.454137
**58**	1	0	-5.651983	2.359391	-5.183006
**59**	1	0	-1.610388	6.928857	-1.907422
**60**	1	0	0.226708	-6.197403	-5.631061
**61**	1	0	1.117881	-1.941103	-1.940398
**62**	1	0	3.786989	-1.869547	-6.85817
**63**	1	0	0.023609	1.053676	-8.361192
**64**	1	0	-4.138363	-0.808564	-7.215548
**65**	1	0	-2.680369	-7.795841	-2.550587
**66**	1	0	0.318912	-7.35202	-1.1388
**67**	1	0	1.484728	2.045682	-4.085076
**68**	1	0	4.793524	-3.585975	-1.771731
**69**	1	0	-0.748885	-4.114729	-9.081296
**70**	1	0	-3.712451	-3.909245	-0.72463

**YST-MN-4b000004_en_**		**Standard Orientation (Ångstroms)**			
**Center number**	Atomic number	Atomic Type	X	Y	Z
**0**	6	0	8.245142	0.005044	5.021731
**1**	6	0	5.848303	-0.78494	6.410767
**2**	6	0	4.35523	1.478172	7.368597
**3**	6	0	3.864582	3.425003	5.288647
**4**	6	0	6.262614	4.145806	3.913202
**5**	6	0	7.778554	1.892274	2.911406
**6**	8	0	2.11006	2.416315	3.445424
**7**	6	0	-0.383808	2.456063	4.093946
**8**	6	0	-1.93496	1.371524	2.078099
**9**	6	0	-4.394395	0.939735	2.485553
**10**	6	0	-6.261612	-0.116066	0.78246
**11**	6	0	-6.456106	0.361226	-1.824811
**12**	6	0	-8.073843	-1.688629	1.90214
**13**	6	0	-9.932067	-2.859335	0.509947
**14**	6	0	-10.110212	-2.395388	-2.083724
**15**	6	0	-8.396001	-0.764206	-3.189988
**16**	8	0	-1.14575	3.292406	6.094419
**17**	6	0	6.451865	0.672722	0.643906
**18**	8	0	10.212501	2.719321	2.062699
**19**	8	0	5.725796	-1.513576	0.588108
**20**	8	0	6.253393	2.282262	-1.269173
**21**	8	0	6.390428	-2.471882	8.422955
**22**	8	0	5.814934	2.636818	9.337005
**23**	8	0	-11.948346	-3.472023	-3.507278
**24**	8	0	-11.714802	-4.465617	1.478912
**25**	6	0	-4.72572	2.074216	-3.296803
**26**	6	0	-4.172847	4.594272	-2.024514
**27**	8	0	-5.778671	5.84172	-1.000351
**28**	8	0	-1.764904	5.391256	-2.349585
**29**	8	0	-2.418675	0.916313	-4.089004
**30**	6	0	-0.791496	-4.726637	-4.350166
**31**	6	0	1.401068	-2.990762	-3.678883
**32**	6	0	2.043944	-1.150555	-5.827462
**33**	6	0	-0.203402	-0.678516	-7.601114
**34**	6	0	-2.628223	-0.880295	-6.082282
**35**	8	0	-2.997129	-3.355024	-5.226951
**36**	6	0	-1.605794	-6.393808	-2.157359
**37**	8	0	2.949222	1.22403	-4.847853
**38**	8	0	3.458279	-4.549216	-3.005117
**39**	8	0	-0.24794	-2.3665	-9.679909
**40**	8	0	-1.940052	-5.086914	0.158569

**41**	1	0	9.17318	-1.668624	4.277236
**42**	1	0	9.538672	0.90153	6.349636
**43**	1	0	4.641015	-1.829437	5.117789
**44**	1	0	2.55759	0.813803	8.120329
**45**	1	0	3.003597	5.087492	6.144965
**46**	1	0	5.829371	5.456265	2.387052
**47**	1	0	7.48594	5.116836	5.251864
**48**	1	0	-1.012791	0.869314	0.334982
**49**	1	0	-5.074869	1.363528	4.373782
**50**	1	0	-7.968306	-2.032646	3.917961
**51**	1	0	-8.620704	-0.378386	-5.18383
**52**	1	0	9.953882	3.786095	0.604011
**53**	1	0	5.196059	1.559534	-2.661645
**54**	1	0	7.095442	-1.451138	9.764049
**55**	1	0	4.663429	3.437854	10.494188
**56**	1	0	-12.968342	-4.52298	-2.414413
**57**	1	0	-11.412898	-4.72833	3.254786
**58**	1	0	-5.760039	2.643871	-4.999168
**59**	1	0	-1.68223	7.044242	-1.557775
**60**	1	0	-0.173471	-5.994639	-5.86721
**61**	1	0	0.808156	-1.85135	-2.065032
**62**	1	0	3.583478	-1.939015	-6.933591
**63**	1	0	-0.025668	1.218926	-8.379965
**64**	1	0	-4.278724	-0.448121	-7.24006
**65**	1	0	-3.42711	-7.22424	-2.635442
**66**	1	0	-0.233365	-7.919892	-1.96337
**67**	1	0	1.493825	2.076162	-4.126941
**68**	1	0	4.496064	-3.579187	-1.827888
**69**	1	0	-1.249908	-3.816552	-9.212996
**70**	1	0	-0.294379	-4.897354	0.915864

YST-MN-4b000004_tddft_		Standard Orientation (Ångstroms)			
**Center number**	Atomic number	Atomic Type	X	Y	Z
**0**	6	0	8.245142	0.005044	5.021731
**1**	6	0	5.848303	-0.78494	6.410767
**2**	6	0	4.35523	1.478172	7.368597
**3**	6	0	3.864582	3.425003	5.288647
**4**	6	0	6.262614	4.145806	3.913202
**5**	6	0	7.778554	1.892274	2.911406
**6**	8	0	2.11006	2.416315	3.445424
**7**	6	0	-0.383808	2.456063	4.093946
**8**	6	0	-1.93496	1.371524	2.078099
**9**	6	0	-4.394395	0.939735	2.485553
**10**	6	0	-6.261612	-0.116066	0.78246
**11**	6	0	-6.456106	0.361226	-1.824811
**12**	6	0	-8.073843	-1.688629	1.90214
**13**	6	0	-9.932067	-2.859335	0.509947
**14**	6	0	-10.110212	-2.395388	-2.083724
**15**	6	0	-8.396001	-0.764206	-3.189988
**16**	8	0	-1.14575	3.292406	6.094419
**17**	6	0	6.451865	0.672722	0.643906
**18**	8	0	10.212501	2.719321	2.062699
**19**	8	0	5.725796	-1.513576	0.588108
**20**	8	0	6.253393	2.282262	-1.269173
**21**	8	0	6.390428	-2.471882	8.422955
**22**	8	0	5.814934	2.636818	9.337005
**23**	8	0	-11.948346	-3.472023	-3.507278
**24**	8	0	-11.714802	-4.465617	1.478912
**25**	6	0	-4.72572	2.074216	-3.296803
**26**	6	0	-4.172847	4.594272	-2.024514
**27**	8	0	-5.778671	5.84172	-1.000351
**28**	8	0	-1.764904	5.391256	-2.349585
**29**	8	0	-2.418675	0.916313	-4.089004
**30**	6	0	-0.791496	-4.726637	-4.350166
**31**	6	0	1.401068	-2.990762	-3.678883
**32**	6	0	2.043944	-1.150555	-5.827462
**33**	6	0	-0.203402	-0.678516	-7.601114
**34**	6	0	-2.628223	-0.880295	-6.082282
**35**	8	0	-2.997129	-3.355024	-5.226951
**36**	6	0	-1.605794	-6.393808	-2.157359
**37**	8	0	2.949222	1.22403	-4.847853
**38**	8	0	3.458279	-4.549216	-3.005117
**39**	8	0	-0.24794	-2.3665	-9.679909
**40**	8	0	-1.940052	-5.086914	0.158569

**41**	1	0	9.17318	-1.668624	4.277236
**42**	1	0	9.538672	0.90153	6.349636
**43**	1	0	4.641015	-1.829437	5.117789
**44**	1	0	2.55759	0.813803	8.120329
**45**	1	0	3.003597	5.087492	6.144965
**46**	1	0	5.829371	5.456265	2.387052
**47**	1	0	7.48594	5.116836	5.251864
**48**	1	0	-1.012791	0.869314	0.334982
**49**	1	0	-5.074869	1.363528	4.373782
**50**	1	0	-7.968306	-2.032646	3.917961
**51**	1	0	-8.620704	-0.378386	-5.18383
**52**	1	0	9.953882	3.786095	0.604011
**53**	1	0	5.196059	1.559534	-2.661645
**54**	1	0	7.095442	-1.451138	9.764049
**55**	1	0	4.663429	3.437854	10.494188
**56**	1	0	-12.968342	-4.52298	-2.414413
**57**	1	0	-11.412898	-4.72833	3.254786
**58**	1	0	-5.760039	2.643871	-4.999168
**59**	1	0	-1.68223	7.044242	-1.557775
**60**	1	0	-0.173471	-5.994639	-5.86721
**61**	1	0	0.808156	-1.85135	-2.065032
**62**	1	0	3.583478	-1.939015	-6.933591
**63**	1	0	-0.025668	1.218926	-8.379965
**64**	1	0	-4.278724	-0.448121	-7.24006
**65**	1	0	-3.42711	-7.22424	-2.635442
**66**	1	0	-0.233365	-7.919892	-1.96337
**67**	1	0	1.493825	2.076162	-4.126941
**68**	1	0	4.496064	-3.579187	-1.827888
**69**	1	0	-1.249908	-3.816552	-9.212996
**70**	1	0	-0.294379	-4.897354	0.915864

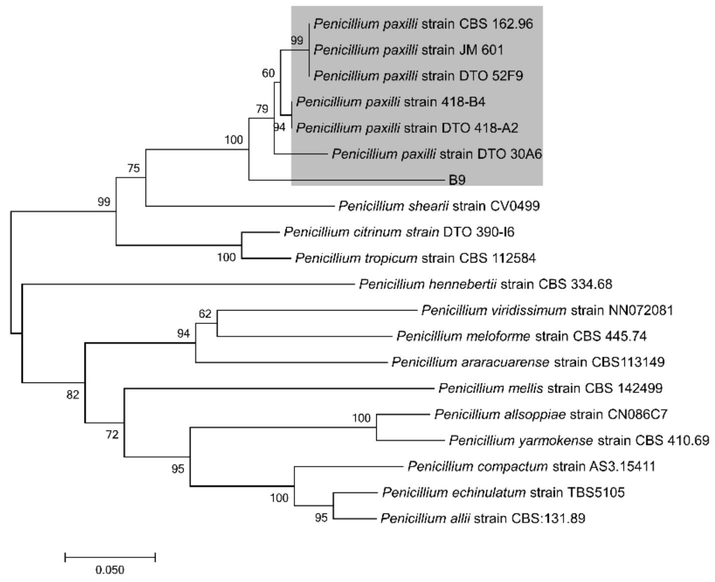

Figure S12. Phylogenetic tree of strain B9 based on the CaM sequence; the evolutionary history was inferred using the neighbor-joining method. Phylogenetic analyses were conducted with 1,000 bootstrap replications in MEGA7. Numbers above branches indicate bootstrap values.
